# High Genetic and Epigenetic Stability in *Coffea arabica* Plants Derived from Embryogenic Suspensions and Secondary Embryogenesis as Revealed by AFLP, MSAP and the Phenotypic Variation Rate

**DOI:** 10.1371/journal.pone.0056372

**Published:** 2013-02-13

**Authors:** Roberto Bobadilla Landey, Alberto Cenci, Frédéric Georget, Benoît Bertrand, Gloria Camayo, Eveline Dechamp, Juan Carlos Herrera, Sylvain Santoni, Philippe Lashermes, June Simpson, Hervé Etienne

**Affiliations:** 1 Unité Mixte de Recherche Résistance des Plantes aux Bioagresseurs, Centre de Coopération Internationale en Recherche Agronomique pour le Développement, Montpellier, France; 2 Unité Mixte de Recherche Résistance des Plantes aux Bioagresseurs, Institut de Recherche pour le Développement, Montpellier, France; 3 Centro Nacional de Investigaciones de Café, Manizales, Colombia; 4 Unité Mixte de Recherche Amélioration Génétique et Adaptation des Plantes Tropicales et Méditerranéennes, Institut National de la Recherche Agronomique, Montpellier, France; 5 Department of Plant Genetic Engineering, Centro de Investigación y de Estudios Avanzados del Instituto Politécnico Nacional, Irapuato, Guanajuato, Mexico; Nanjing Agricultural University, China

## Abstract

Embryogenic suspensions that involve extensive cell division are risky in respect to genome and epigenome instability. Elevated frequencies of somaclonal variation in embryogenic suspension-derived plants were reported in many species, including coffee. This problem could be overcome by using culture conditions that allow moderate cell proliferation. In view of true-to-type large-scale propagation of *C. arabica* hybrids, suspension protocols based on low 2,4-D concentrations and short proliferation periods were developed. As mechanisms leading to somaclonal variation are often complex, the phenotypic, genetic and epigenetic changes were jointly assessed so as to accurately evaluate the conformity of suspension-derived plants. The effects of embryogenic suspensions and secondary embryogenesis, used as proliferation systems, on the genetic conformity of somatic embryogenesis-derived plants (emblings) were assessed in two hybrids. When applied over a 6 month period, both systems ensured very low somaclonal variation rates, as observed through massive phenotypic observations in field plots (0.74% from 200 000 plant). Molecular AFLP and MSAP analyses performed on 145 three year-old emblings showed that polymorphism between mother plants and emblings was extremely low, i.e. ranges of 0–0.003% and 0.07–0.18% respectively, with no significant difference between the proliferation systems for the two hybrids. No embling was found to cumulate more than three methylation polymorphisms. No relation was established between the variant phenotype (27 variants studied) and a particular MSAP pattern. Chromosome counting showed that 7 of the 11 variant emblings analyzed were characterized by the loss of 1–3 chromosomes. This work showed that both embryogenic suspensions and secondary embryogenesis are reliable for true-to-type propagation of elite material. Molecular analyses revealed that genetic and epigenetic alterations are particularly limited during coffee somatic embryogenesis. The main change in most of the rare phenotypic variants was aneuploidy, indicating that mitotic aberrations play a major role in somaclonal variation in coffee.

## Introduction

**Figure 1:**
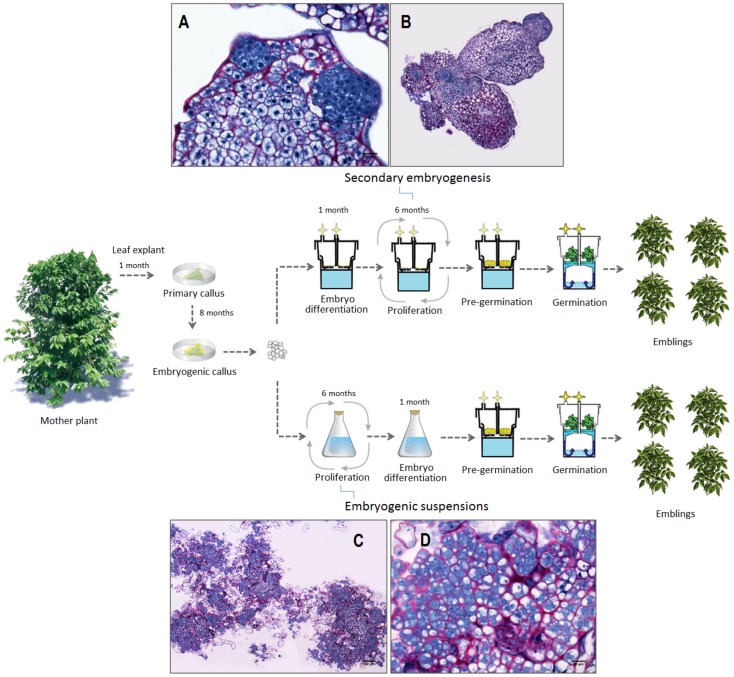
Schematic representation of two somatic embryogenesis processes applied at the industrial level. The first somatic embryogenesis process (upper section of the flow diagram) involved a proliferation step based on secondary embryogenesis in RITA^®^ temporary immersion bioreactors (photos 1A, 1B). The second process (lower section of flow diagram) included a proliferation step based on embryogenic suspensions (photos 1C, 1D). 1A, initial developmental stages of secondary embryos at the root pole of primary somatic embryos; 1B, clusters of primary and secondary embryos; 1C, clusters of embryogenic cells in suspension; 1D, embryogenic cells in suspension.

Among micropropagation methods, somatic embryogenesis has the best potential for rapid and large-scale multiplication of selected varieties in a wide range of economically important species. Schematically, the initial step of dedifferentiation leading to the acquisition of embryogenic competence is common to most plant species, whereas for the following step of proliferation of embryogenic material, efficient procedures can be classified under two main strategies. The first is the proliferation through secondary embryogenesis (SCE) which involves first differentiating the somatic embryos before enhancing their proliferation by adventitious budding (Figure 1). The second consists of establishing embryogenic suspensions (ESP) to favor large-scale embryogenic cell proliferation before the subsequent embryo differentiation step. In order to come up with an industrial procedure, the development of ESP represents the best option to ensure synchronous and massive somatic embryo production [1]. In addition, ESP allows the production of large numbers of embryogenic-competent cells and this process can be easily scaled up. Nevertheless, tissue culture systems such as somatic embryogenesis that involve the acquisition of competence for pluripotentiality and extensive cell division are more risky with respect to genome and epigenome instability [2]. The use of ESP has frequently been associated with an increased risk of genetic instability and somaclonal variation in the regenerated plants [3]–[5]. Although ESP has been developed for some important crops, it has therefore not been widely applied for commercial purposes. Somaclonal variation in ESP-derived plants is probably related to the presence of 2,4-dichlorophenoxyacetic acid (2,4-D), which is often essential for maintaining proliferating cells in an embryogenic, undifferentiated state [6], [7]. This auxin could enhance somaclonal variation through the stimulation of rapid disorganized growth that can influence the mitotic process, resulting in chromosomal aberrations [8], [9].

The term ‘somaclonal variation’ (SV) describes the tissue culture-induced stable genetic, epigenetic or phenotypic variation in clonally propagated plant populations [10]. Somaclonal variation is considered to be one of the main bottlenecks in the development of micropropagation procedures, especially in view of large-scale commercial operations, for which the strict maintenance of genetic and agronomic traits from selected individuals is required. An analysis of the progeny of phenotypic variants showed that some of the variations produced by somatic embryogenesis can occur in the form of stable and heritable mutations [8], [11]. In maize, Kaeppler and Phillips [12] also reported stable segregation of somaclonal variant phenotypic qualities in several seed generations. It has also been well documented that somaclonal variants commonly present cytological aberrations such as chromosomal rearrangements (deletions, duplications, inversions and translocations), and sometimes more severe alterations like aneuploidy or polyploidy [12]–[16].

Although most mutants segregate in a Mendelian fashion upon selfing and outcrossing [12], SV is sometimes present in the form of transient mutations, suggesting the involvement of epigenetic events [11]. Epigenetic traits are heritable changes associated with chemical modification of DNA without alteration of the primary DNA sequence [17]. Cytosine methylation has been proposed as a possible cause of SV [11], [12]. Epigenetic modifications (methylation) can mediate the transmission of an active or silent gene in the short-term (mitotic cell division) or long-term (meiotic divisions leading to transmission across generations) [18]. DNA methylation in plants commonly occurs at cytosine (5-methylcytosine, m^5^C) bases in all sequence contexts: the symmetric CG and CHG (in which H could be A, T or C) and the asymmetric CHH contexts [17], [18]. Molecular marker approaches like methylation-sensitive amplified polymorphism (MSAP) and Met-AFLP have proved efficient in the analysis of methylation patterns [19], [20]. The existence of zones susceptible to methylation variations was recently shown in somatic embryogenesis-derived plants (emblings) in grapevine [21] and barley [20]. SV was also associated with the activity of mobile DNA elements or retroelements [22], [23]. Novel mechanisms such as RNAi directed demethylation have recently been proposed to explain retrotransposon activation [2], [24].

*Coffea arabica* is an allotetraploid tree species (2n = 4X = 44) characterized by low molecular polymorphism [25]. Somatic embryogenesis is currently applied industrially for large-scale and rapid dissemination of selected F1 hybrids that provide a highly significant increase in the yield of high quality coffee [26], [27]. Regarding industrial-scale micropropagation, upgrading production to several million vitroplants per production unit would undoubtedly boost economic profitability. This would require switching from an SCE- to an ESP-based protocol. However, former field observations revealed that SV occurs at relatively high rates in ESP-derived *C. arabica* plants [28], [29]. Apart from different phenotypic variants easily identifiable through morphological characteristics, we did not discover in trees showing a normal phenotype any variations involving agronomically important quantitative and physiological characteristics [30]. In view of true-to-type propagation of selected *C. arabica* hybrid varieties, we previously developed improved ESP protocols based on the use of low exogenous 2,4-D concentrations and short proliferation periods, allowing reliable somatic embryo mass regeneration [27]. For potential commercial applications, here using two *C. arabica* hybrids we verified the conformity of suspension-derived plants with that of plants obtained by secondary embryogenesis, i.e. the industrial process currently in use. The objectives were to assess large-scale phenotype conformity in commercial field plots, to quantify genetic and epigenetic modifications in the regenerated plants through AFLP (Amplified fragment length polymorphism) and MSAP molecular markers, and to cytologically characterize the karyotype of different phenotypic variants detected in the study.

## Results

### Frequency of phenotypic variants

Embling batches of hybrids HI and H3 obtained from both SCE and ESP were checked for phenotype variation at both nursery and field levels. The frequency of phenotypic variants assessed among more than 600 000 plants in the nursery was very low (approx. 0.1%) and not significantly affected by the proliferation system nor the hybrid variety (Table 1). Observation of around 200 000 emblings in the field two years after planting revealed roughly an additional 0.74% of abnormal phenotypes, still without any significant difference between the two proliferation systems and hybrids. Apart from these phenotypic variants, all the other studied trees flowered, grew and produced normally. In conclusion, the overall phenotypic variation rate obtained by pooling the data obtained both in the nursery and in the field was less than 1% and no significant differences were noted between the proliferation systems or between the hybrids used.

**Table 1:** Frequency of coffee phenotypic variants detected in the nursery and field on three year-old plants from two *C. arabica* hybrids depending on the type of proliferation system used in the industrial somatic embryogenesis process.

Proliferation system in the somatic embryogenesis process	Hybrid	Observations after 10 months in nursery				Observations after 36 months in field			
		No. of observed emblings	No. of variants	Somaclonal variation frequency (%)	3σ confidence interval*	No. of emblings	No. of variants	Somaclonal variation frequency (%)	3σ confidence interval*
Secondary embryogenesis	H1	117.115	148	0.13	[0.09–0.16]	51.131	373	0.73	[0.62–0.84]
	H3	121.894	159	0.13	[0.10–0.16]	49.126	390	0.79	[0.67–0.91]
	*Total*	*239.009*	*307*	*0.13*	*[0.10*–*0.15]*	*100.257*	*763*	*0.76*	*[0.67*–*0.84]*
Embryogenic suspension	H1	204.871	206	0.10	[0.08–0.12]	54.218	402	0.74	[0.63–0.85]
	H3	197.705	183	0.09	[0.07–0.11]	54.566	394	0.72	[0.61–0.83]
	*Total*	*402.576*	*389*	*0.09*	*[0.08*–*0.11]*	*108.784*	*796*	*0.73*	*[0.65*–*0.80]*

The variable analyzed was the proportion (p) of variant (p = X/n), where X was the number of variant and n the number of plants observed. A 3σ confidence limit for binomial distribution was calculated using the formula 
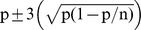
 with a level of confidence of 99%.

**Figure 2:**
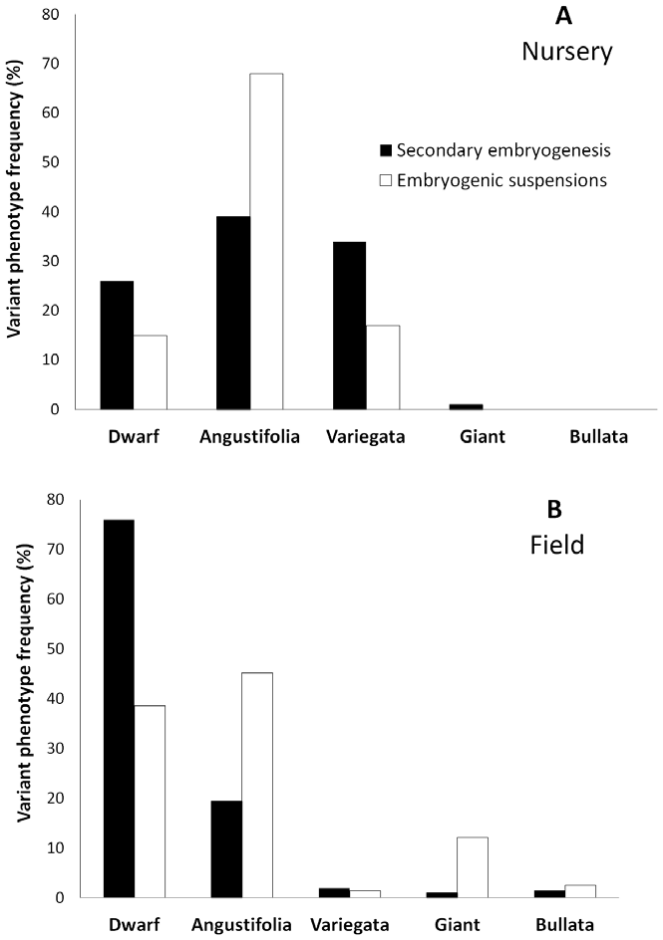
Proportions (%) of the different types of phenotypic variants in comparison to the total number of variants. Variants representing less than 1% of somatic embryogenesis-derived plants were observed in *C. arabica* embling batches at nursery (A) and field (B) levels, depending on the proliferation system used, i.e. secondary embryogenesis (SCE) or embryogenic suspension (ESP). In the nursery, the data were obtained from 239 009 emblings derived from SCE and 402 576 emblings from ESP. In the field, the data were obtained through the observation of 100 257 emblings derived from secondary embryogenesis and 108 784 from embryogenic suspensions.

**Figure 3:**
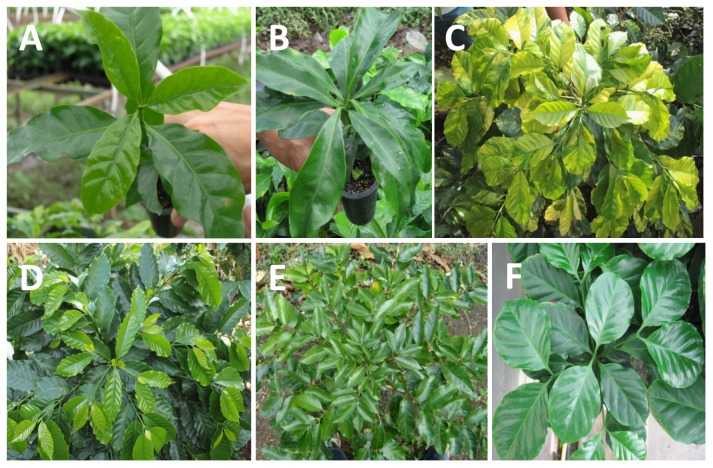
Examples of different *C. arabica* phenotypic variants in plants mass propagated through somatic embryogenesis. A, plant exhibiting a normal phenotype in the nursery; B, Angustifolia variant in the nursery; C, Variegata variant in the field; D, plant showing a normal phenotype in the field; E, Dwarf variant in the field; F, Bullata variant in nursery.

Both proliferation systems generated the same kind of phenotypic variants (Figures 2A, B), with the Dwarf and Angustifolia types (Figures 3E, B) being the most frequent. Note that the secondary embryogenesis proliferation system specifically enhanced the occurrence of Dwarf variants, whereas the embryogenic suspensions favored the occurrence of the Angustifolia type. This latter phenotype can easily be detected and eliminated at the nursery level along with the Variegata variant (Figure 3C). A comparison of Figures 2A and 2B clearly shows that elimination in the nursery is not efficient for the Dwarf type. This somaclonal variation is more easily observable 2–3 years after planting in the field thanks to the characteristic grouped canopy morphology and low yield. Similarly, the Giant and Bullata (Figure 3F) phenotypic variants could only be detected in the field on well-developed trees.

### Locus specific polymorphisms revealed by AFLP

In order to verify the induction of molecular polymorphism by the somatic embryogenesis process, AFLP analysis (four primer combinations, Table 2) was carried out on mother plants and their derived emblings. From a total of 204 bands obtained, only one polymorphic fragment of 173 bp in size (*Eco*-ACT/*Mse*-AGT) shared by two emblings of hybrid H1 and exhibiting a normal phenotype, was found (Table 3). From a total of 198 bands obtained, no polymorphism was found in emblings of hybrid H3. All variants had the same AFLP pattern as the mother plants. For both hybrids, no significant quantitative effect on AFLP was detected when comparing SCE and ESP.

**Table 2:** Primer combinations used for AFLP and MSAP analyses.

AFLP primer combinations (*Eco*+3 ^labeled^/*Mse*+3)	MSAP primer combinations (*Eco*−3/*Hpa*+2 ^labeled^)
*Eco*-ACT/*Mse*-AGT	C1 *Eco*-AAC/*HPA*-AA
*Eco*-AGG/*Mse*-AGT	C2 *Eco*-AAC/*HPA*-AT
*Eco*-CGC/*Mse*-CCA	C3 *Eco*-AGG/*HPA*-AA
*Eco*-CAC/*Mse*-CCA	C4 *Eco*-AGG/*HPA*-AT
	C5 *Eco*-ACT/*HPA*-CA
	C6 *Eco*-ACT/*HPA*-CT
	C7 *Eco*-AGA/*HPA*-CA
	C8 *Eco*-AGA/*HPA*-CT

Fluorescent dyes for marked primers correspond to 5'-FAM ^TM^ and 5'-HEX ^TM^

**Table 3:** Summary of AFLP data and observed polymorphisms among mother plants and emblings derived from secondary embryogenesis or embryogenic suspensions.

Proliferation system in the somatic embryogenesis process	Hybrid	No. of analyzed emblings	No. of fragments	Polymorphic fragments		Emblings showing polymorphisms		Total polymorphism **
				No.	(%)	No.	(%)	(%)
Secondary embryogenesis	H1	33	204	1 *	0.5	2	6	0.03
	H3	45	198	0	0	0	0	0
Embryogenic suspensions	H1	26	204	0	0	0	0	0
	H3	41	198	0	0	0	0	0

Data were obtained for two *C. arabica* hybrids (H1 and H3) and compared with the patterns of the mother plants.

* Found in 2 emblings with normal phenotype (N°210 and 232) showing a new AFLP band Eco-ACT/Mse-AGT 173 bp

** Total polymorphism  =  [No. of polymorphic fragments/(No. of fragments × No. emblings)]×100

### Methylation changes revealed by MSAP

**Figure 4:**
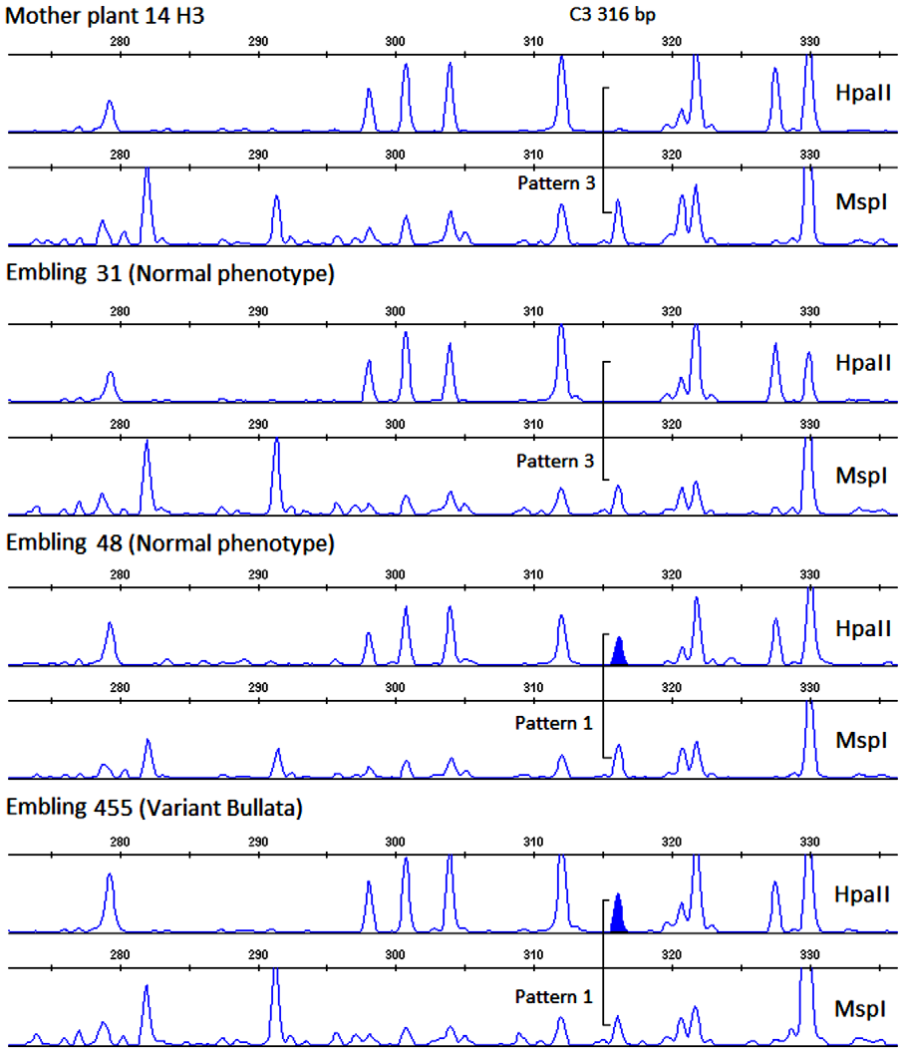
Representation of MSAP electropherograms observed for coffee mother plants and embling progeny using the isoschizomers *Hpa*II and *Msp*I. Illustration of the pattern variation obtained for normal and variant phenotypes within the embling progeny.

In order to evaluate the occurrence of possible epigenetic modifications in the micropropagated plants, a study on the alteration of methylation patterns was performed by MSAP analysis using eight primer combinations (Table 2). Only clear and reproducible bands were selected for the analysis. More than 395 fragments were considered. First, MSAP patterns were obtained from DNA digested by the two isoschizomers (*Hpa*II and *Msp*I), as illustrated in Figure 4. They were further compared with those of mother plants to classify the amplified fragments according to the methylation pattern, as shown in Table 4. The percentages of monomorphic fragments (pattern 1) were elevated and similar for both hybrids at nearly 91%. The remaining 9% of fragments (8.5% for H1 and 9.1% for H3) almost exclusively corresponded to pattern 3.

**Table 4:** MSAP patterns corresponding to different methylation states of the symmetric sequence CCGG, as revealed by the specificity of the restriction enzymes *Hpa* II and *Msp* I.

Restriction enzymes	MSAP patterns after enzymatic digestion			
	Pattern 1	Pattern 2	Pattern 3	Pattern 4
*Hpa* II	1	1	0	0
*Msp* I	1	0	1	0
Methylation state	Unmethylated	Hemi-methylated	Fully-methylated	Fully-methylated
Methylation position	None	External cytosine	Internal cytosine	External cytosine
Schematic representation	CCGGGGCC	_CH3_CCGGGGCC	_CH3_CCGGGGCCCH3	_CH3_CCGGGGCCCH3

A comparison of amplification patterns in mother plants and their respective progeny are reported in Table 5. All differences between mother and derived plants were switches between patterns 1 and 3 and vice versa, i.e. likely modifications in the restriction ability of *Hpa*II. Among the polymorphic bands, eight bands corresponded to a change from pattern 3 in mother plants towards the unmethylated pattern 1 in emblings, suggesting demethylation. Changes associated with certain polymorphic MSAP bands were more frequent than others (Table 5). Seventy percent of the changes were linked to only five polymorphic bands. The detected polymorphism was very low and similar for both hybrids (Table 6) but slightly higher (0.07–0.18%) when compared to AFLP molecular markers. Similar to the AFLP results, the total MSAP polymorphism was not significantly different between the two proliferation systems nor between the two hybrid varieties.

**Table 5:** MSAP methylation patterns in mother plants and modified patterns in emblings.

Polymorphic MSAP fragments (size in bp)	MSAP methylation patterns		Proliferation system affected by the methylation change		Presence of the methylation change depending on the plant phenotype		No. of methylation changes for each fragment	
	Mother plants	Emblings	Hybrid H1	Hybrid H3	Normal	Variant	No.	(%)
								
C3- 107 bp	Pattern 3	Pattern 1	SCE, ESP	SCE, ESP	+**	−	8	9.8
C2 -127 bp	Pattern 3	Pattern 1	0	ESP	+	−	1	1.2
C4 -134 bp	Pattern 3	Pattern 1	0	SCE	+	−	4	4.8
C1- 251 bp	Pattern1*	Pattern 3	SCE, ESP	0	+	−	12	14.6
C3- 253 bp	Pattern 3	Pattern 1	0	SCE	+	−	2	2.4
C2- 302 bp	Pattern 3	Pattern 1	0	SCE, ESP	+	+	16	19.5
C3- 316 bp	Pattern 3	Pattern 1	0	SCE	+	+	4	4.8
C6 -370 bp	Pattern 3	Pattern 1	SCE, ESP	ESP	+	+	10	12.2
C8- 370 bp	Pattern 3	Pattern 1	SCE, ESP	SCE, ESP	+	+	12	14.6
C5- 387 bp	Pattern 1	Pattern 3	SCE	SCE	+	−	13	15.8
No. changes							82	

Relation with the type of *C. arabica* hybrid, type of proliferation system [secondary embryogenesis (SCE) and embryogenic suspension (ESP)] and regenerant phenotype.

* Pattern 1: Fragment present in both HpaII and MspI restriction digests (1∶1); Pattern 3: Fragment absent in HpaII digests and present in MspI digests (0∶1). ** Relationship with a particular phenotype is indicated with (+) for presence and (−) for absence.

**Table 6:** Overall MSAP data and methylation polymorphism among mother plants and emblings of *C. arabica* hybrids derived from secondary embryogenesis or embryogenic suspensions.

Proliferation in the somatic embryogenesis process	Hybrid	No. of emblings analyzed	No. of fragments	Methylation polymorphic fragments		Total polymorphism*(%)	3σ confidence intervals**
				No.	Percentage (%)		
Secondary embryogenesis	H1	33	399	5	1.2	0.18	[0.071–0.294]
	H3	45	396	7	1.7	0.16	[0.068–0.246]
		78		12	1.5	0.17	[0.098–0.238]
Embryogenic suspensions	H1	26	399	4	1.0	0.18	[0.057–0.309]
	H3	41	396	5	1.0	0.07	[0.006–0.129]
		67		9	1.1	0.11	[0.051–0.174]

* Total polymorphism  =  [No. of methylation polymorphic fragments/(No. of fragments × No. emblings)]×100.

** The variable analyzed was the proportion (p) of methylation polymorphisms (p = X/n), where X was the number of methylation polymorphisms and n the total number of fragments. A 3σ confidence limit for binomial distribution was calculated using the formula 
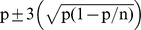
 with a level of confidence of 99%.

**Figure 5:**
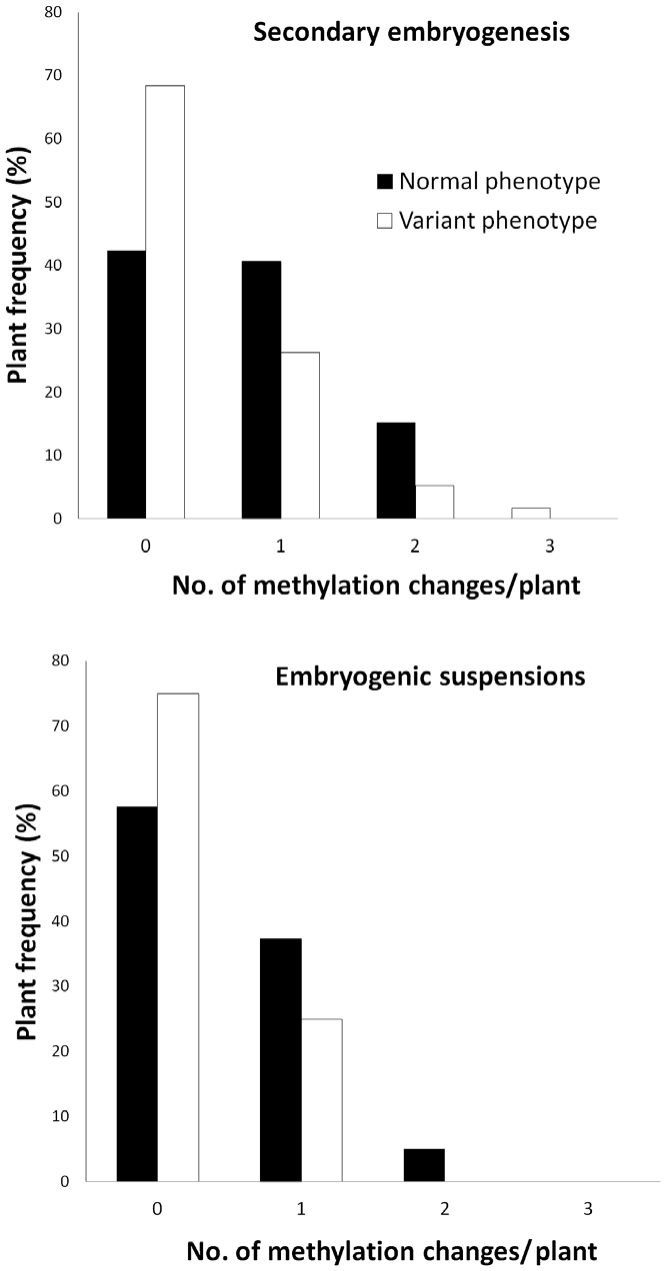
Methylation polymorphism accumulation in coffee emblings showing a normal *vs*. variant phenotype depending on the somatic embryogenesis process used. For the secondary embryogenesis process, data were derived from the analysis of 59 phenotypically normal and 19 variant emblings. For the embryogenic suspension process, 59 phenotypically normal and 8 variant emblings were studied.

Most emblings showing changes in MSAP pattern had only one or two methylation polymorphisms (Figure 5). We did not find any emblings with more than three methylation polymorphisms. The same polymorphic bands were shared by plants from both proliferation systems and/or both hybrids in approximately half of the cases (Table 5). No relation was established between the variant phenotype and a particular MSAP pattern (Table 6).

### Chromosome counting of somaclonal variants

**Figure 6:**
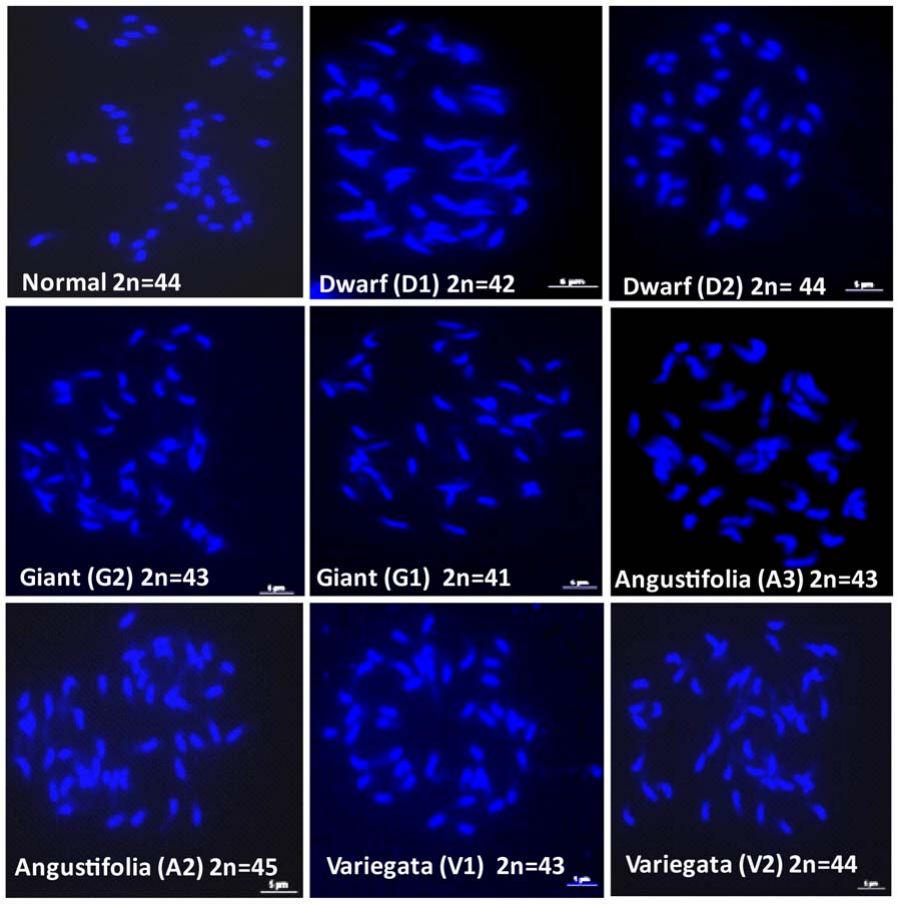
Mitotic cells at metaphase or prometaphase stages and observed ploidy levels of some normal and variant emblings from the allotetraploid *C. arabica* species (2n = 44). Karyotype analyses were performed by counting chromosomes on four to eight clear metaphase spreads obtained from root tips of three year-old plants.

Chromosome numbers were assessed in 2 phenotypically normal emblings and 11 somaclonal variants (Fig. 6). Table 7 shows that the two normal regenerants exhibited the expected chromosome number for the allotetraploid *C. arabica* species (2n = 4x = 44) whereas 7 of the 11 variant emblings showed a different chromosome number (aneuploids). In almost all cases, the aneuploid karyotypes were characterized by the loss of 1–3 chromosomes. One Angustifolia variant had an extra chromosome. Moreover, the results showed that different chromosome numbers - including the normal number - could be observed for the same variant phenotype and that abnormal chromosome numbers were obtained for most of the variant phenotypes.

**Table 7:** Summary of chromosome counting in some normal *versus* variant *C. arabica* hybrids derived from somatic embryogenesis.

Embling phenotype (normal or variant)	Somatic embryogenesis process	Code	No. of metaphases analyzed	No. of chromosomes
Normal	ESP	N1	8	44
Normal	SCE	N2	7	44
Angustifolia	ESP	A1	4	44
Angustifolia	SCE	A2	8	45
Angustifolia	ESP	A3	8	43
Bullata	ESP	B1	4	44
Dwarf	SCE	D1	5	42
Dwarf	ESP	D2	4	44
Giant	ESP	G1	6	41
Giant	ESP	G2	6	43
Giant	SCE	G3	6	43
Variegata	ESP	V1	6	43
Variegata	SCE	V2	4	44

The chromosome numbers obtained from root tips are indicated for 3 year-old emblings derived from embryogenic suspensions (ESP) or secondary embryogenesis (SCE) showing normal or abnormal phenotypes.

## Discussion

Until now, relatively high somaclonal variation rates have been reported in *C. arabica* emblings, particularly with embryogenic suspensions [28], [29], [30]. The variant frequency was found to strongly increase in embryogenic suspensions after 6 months proliferation (25% after 12 months) in the presence of 4.52 μM 2,4-D [29]. The presence of disorganized rapid growth phases in tissue culture, such as callus and cell suspension cultures, has often been considered as one of the factors that cause SV [8], [31]. In view of true-to-type propagation, we further established processes with limited disorganized rapid growth phases. The first was based on SCE proliferation in temporary immersion bioreactors enhanced by the addition of cytokinin, similar to methods described in other woody plants such as rubber [32], oak [33] and tea [34] and is currently used on a commercial scale for coffee. The second involves the proliferation of ESP in the presence of both auxin and cytokinin albeit at a very low level of 2,4-D (1.36 µM) with a short proliferation time (6 months). These conditions allow sufficient amplification of embryogenic material to ensure the cost-effectiveness of the industrial process. A large-scale phenotypic evaluation for each process: 230 000–400 000 emblings in the nursery and 100 000 in the field was performed to obtain valuable and accurate information on genetic stability. The present study showed for the first time that, for both somatic embryogenesis processes, the variant phenotype frequencies were extremely low (less than 1%) and not statistically different. Moreover no statistical difference could be noted between the two studied hybrids. This clearly demonstrates that it is possible to control SV by optimizing the ESP culture conditions. In another cultivated *Coffea* species, i.e. the diploid *C. canephora*, Ducos *et al*. [35] found 2–4% of a low-vigor phenotype off-type among 5 067 emblings derived from 5–7-month-old ESP. In contrast to the *C. arabica* protocol, all somatic embryogenesis steps for *C. canephora* were performed with cytokinins as sole source of growth regulator. Hence, in coffee, both auxin and cytokinin could probably be involved in the generation of SV. The Angustifolia, Variegata and Dwarf variants were the most frequent types, irrespective of the proliferation strategy employed, confirming previous studies conducted on a lesser scale [29], [30]. Interestingly, except for the Dwarf variant which seems to be tissue culture specific, all the phenotypic variants observed in the somatic embryogenesis progenies were also observed among *C. arabica* seed-derived progeny. Indeed, phenotypic mutations are frequent in coffee nurseries, and Krug and Carvalho [36] previously characterized the numerous different morphologies in detail.

In several cases, AFLP markers have proved useful in the detection of genetic variation in tissue culture derived plants [20], [37]–[40]. In coffee, the genetic stability of coffee emblings has been poorly evaluated by molecular markers and limited to plants derived from experimental protocols. The present study revealed no or extremely limited mutations at the DNA level in large-scale somatic embryogenesis-propagated plants**.** From a total sample of 145 plants analyzed belonging to two hybrid varieties, only 1 out of 204 bands was polymorphic in only two SCE-produced plants. ESP-derived plants showed no AFLP polymorphism when compared with mother plants. Our results significantly differed from those previously reported in *C. arabica*. In a first approach on only 27 plants, Rani *et al.* [41] used different DNA markers (RAPD, random amplified polymorphic DNA and SSR, simple sequence repeat) to assess the genetic integrity of *C. arabica* emblings obtained from embryogenic calli, and they found a higher polymorphism level (4%) in the nuclear genome. By performing RAPD analyses on Norway spruce emblings, Heinze and Schmidt [42] concluded that gross somaclonal variation was absent in their plant regeneration system. In contrast, RAPD and SSR markers allowed the detection of high genetic variation in cotton emblings regenerated in the presence of 2,4-D [39]. AFLP analysis of 24 rye emblings led to the scoring of 887 AFLP markers, among which 8.8% identified the same polymorphism in plants obtained independently, revealing putative mutational hot spots [38].

DNA cytosine methylation plays an important role in plant regulation and development [43], [44]. Since the pioneer studies on maize emblings [12], substantial evidence has been obtained which indicates that demethylation can occur at a high frequency during somatic embryogenesis and can be an important cause of tissue culture induced variation [45]. DNA methylation has also been implicated in gene silencing and transposable element reactivation [24], [46]. To our knowledge, epigenetic deregulation during coffee micropropagation has not yet been studied. The very low total methylation polymorphism values obtained for both somatic embryogenesis processes and both hybrids (0.07–0.18% range) indicated that the tissue culture procedures employed in coffee weakly affected DNA methylation of the regenerated plants. This finding is in accordance with the 0.87% total polymorphism recently found in *Freesia hybrid* emblings by Gao *et al.* [40]. Moreover, the low number of methylation polymorphisms per embling (range 1–3) confirmed that neither SCE nor ESP induced additional stress at the methylation level during embryogenic material proliferation. In contrast, a significantly higher accumulation of methylation changes in some emblings has been regularly observed in other species [20], [21], [47], [48]. For example, in grapevine, most emblings showed between zero and three changes, similar to our findings, but a few accumulated up to 18 [21]. It has also been well demonstrated that auxin levels strongly alter DNA methylation of embryogenic cell cultures [49]. However, similar to our results, examples of stable MSAP patterns have already been reported using bamboo tissues at different developmental stages of somatic embryogenesis [50]. The timing of plant regeneration from proliferating callus cultures could be crucial for the appearance of variation. In callus-derived hop plants, an increase in the variation was detected by MSAP in prolonged callus cultures [20]. Our results demonstrated that very few changes are possible by limiting both the auxin level and culture duration.

MSAP markers have already been successfully used to demonstrate epigenetic instabilities (methylation alteration) induced by somatic embryogenesis in a great variety of plants, such as the ornamental flower *Freesia*, banana, barley, grapevine and maize [20], [21], [40], [51]–[53], also indicating that demethylation events were generally the most frequent. Although occurring at low frequency, our results also indicated demethylation events and mainly the loss of methylation in the internal cytosine of the 5'-CCGG-3′ sequence to produce a new *Hpa*II band not detected in mother plants but present in the amplification pattern of the isoschizomer *Msp*I. The detection of the same MSAP polymorphic fragments in independent plant samples from different hybrids and somatic embryogenesis processes suggests the existence of hotspots of DNA methylation changes in the genome. The existence of non-randomly behaving methylation polymorphic fragments in micropropagated plants has already been described using Met-AFLP [20], [48] and MSAP [21], [53]–[55].

Gross changes, such as variation in ploidy level, number of chromosomes and structural changes, are mitotic aberrations that represent major genomic alterations of *in vitro* plants often generated during *in vitro* proliferation and differentiation [12], [56]–[58]. Variations in chromosome number and structure have been described among emblings for several species [59]–[62]. We demonstrated that gross changes occur during coffee somatic embryogenesis and are related to SV, whereas genetic and epigenetic (methylation) alterations are very weak. Until now, by using flow cytometry analysis, normal ploidy levels were reported in coffee emblings [29], [63] but chromosome counting was not performed in these studies. The sensitivity of flow cytometry analysis was probably not sufficient to identify aneuploid plants. Nevertheless, the presence of mitotic aberrations, including double prophase, lagging chromosomes, aneuploids and polyploid cells, has previously been described in leaves [64], [65] and embryogenic calli [64] of *C. arabica* but not in the later steps of somatic embryogenesis, and without establishing any relation with SV. The presence of aneuploidy has also been well documented in embryogenic calli of *Hordeum vulgare* [66] and sweet orange [60].

The mechanisms underlying SV remain largely theoretical and unclear [16]. Thus it is often difficult to correlate a well-described genetic or epigenetic mechanism to a variant phenotype. For example, although DNA methylation has often been suggested as a possible cause of SV, a number of studies have reported high levels of methylation variation with no effect on the plant phenotype [20], [21], [52]. Another example is given by oil palm emblings, approximately 5% of which exhibit the ‘mantled’ phenotype affecting the formation of floral organs in both male and female flowers. Although a decrease in DNA methylation was observed, it was not possible to determine the nature of the epigenetic deregulation [67]. In the present study, it was possible to reveal a large proportion of aneuploid karyotypes in different variant phenotypes, hence showing that chromosomal rearrangements could be directly involved in the occurrence of phenotypic variation. The addition or subtraction of a single chromosome has a greater impact on phenotype than whole genome duplication, i.e. polyploidy [68]. It was clearly demonstrated in *Arabidopsis thaliana* that certain phenotypic traits are strongly associated with the dosage of specific chromosomes and that chromosomal effects can be additive [69]. Similarly, in maize seedlings, trisomic plants showed characteristic features such as reduced stature, tassel morphology changes and the presence of knots on the leaves, suggesting a phenotypic effect caused by the altered copy of specific chromosome related genes [70]. A similar mechanism could explain most of the variant phenotypes observed in *C. arabica*. The observation of a variant phenotype in plants with the expected chromosome number could be explained by the coexistence of monosomic and trisomic chromosomes in the same genome or by other chromosomal-like structural changes associated with undetected deletions, duplications, inversions or translocations of specific chromosomal segments [10], [71]. The karyotype analyses performed in the present study were limited to chromosome counting and did not enable observation of such chromosomal alterations.

## Conclusions

This report shows that somatic embryogenesis is reliable for true-to-type and large-scale propagation of elite varieties in the *C. arabica* species. Both ESP and SCE ensured high proliferation rates along with very low SV rates, as observed through massive phenotypic observations in a commercial nursery and field plots. Molecular analysis (AFLP and MSAP) performed on 145 emblings derived from two proliferation processes and two different F1 hybrids showed that polymorphism between mother plants and emblings was extremely low. Consequently, it can be concluded that genetic and epigenetic alterations were also particularly limited during somatic embryogenesis in our controlled culture conditions. *C. arabica* is a young allopolyploid still having the most of its genes in duplicated copies [72]. It could be hypothesized that the impact of genetic or epigenetic variations on phenotype was restricted because of the buffer effect due to polyploidy. The main change in most of the rare phenotypic variants was aneuploidy. Although further studies are necessary for an accurate understanding of the chromosome anomalies involved in the acquisition of a particular phenotype, it is now obvious that mitotic aberrations play a major role in SV in coffee. The identification and use of molecular markers at the heterozygous state in mother plants (i.e. polymorphic between the two parental lines) would be required to further investigate this type of chromosome abnormality. Current studies based on the use of long-term embryogenic cultures [73] are aimed at establishing the full range of cytological, genetic and epigenetic (with a special focus on transposable elements) mechanisms underlying SV.

## Materials and Methods

### Plant material and somatic embryogenesis

Selected F1 hybrids of *C. arabica* [26] obtained by crossing traditional dwarf American varieties (Caturra, Sarchimor T5296) and wild accessions originating from Ethiopia and Sudan are disseminated in Central America through somatic embryogenesis. In the present study, emblings derived from the two hybrids Sarchimor T5296 x Rume Sudan and Caturra x ET531, named respectively H1 and H3, were analyzed to assess the SV level. Large-scale phenotypic observations were performed in Nicaragua both at the nursery (more than 600 000 young emblings) and field level (more than 200 000 three year-old emblings) on 11 coffee plots belonging to the ‘La Cumplida’ coffee research experimental sites in the Matagalpa region (Nicaragua). Nursery and field phenotypic observations were done on balanced amounts of plants from hybrids H1 and H3 (Table 1). Field observations were performed for all trees by visual evaluation of growth and morphology, flowering and fruit yield during the first and second production years. No specific permits were required for the described field studies that were performed with the authorization of the Coffee Research Department of ‘La Cumplida’, owner of the experimental sites. These sites are not protected and the studies did not involve endangered or protected species. Molecular marker analysis was applied on F1 hybrid mother plants propagated by rooted horticultural cuttings (four for each hybrid) and used as source material for *in vitro* propagation, as well as on adult emblings (3 years after planting) for which plants exhibiting an abnormal phenotype (phenotypic variant) were distinguished from plants with a normal phenotype and productivity (Table 8). Molecular marker patterns obtained for emblings were systematically compared with those obtained with mother plants (four plants per hybrid propagated by horticultural cutting) used as reference.

**Table 8:** Plant material used in molecular marker analyses.

Somatic embryogenesis proliferation step	Type of plant material	No. of plants per *C. arabica* hybrid		Total no. of plants
		H1	H3	
Secondary embryogenesis	Emblings normal phenotype	28	31	59
	Emblings variant phenotype	5	14	19
	Angustifolia (A)	0	4	
	Bullata (B)	2	4	
	Dwarf (D)	0	2	
	Variegata (V)	3	4	
	Total	33	45	78
Embryogenic suspension	Emblings normal phenotype	25	34	59
	Emblings variant phenotype	1	7	8
	Angustifolia (A)	1	2	
	Dwarf (D)	0	1	
	Variegata (V)	0	4	
	Total	26	41	67

Total number of plants analyzed from two F1 *Coffea arabica* hybrid lines (H1 and H3) corresponding to somatic-embryo derived 3 year-old plants (emblings) with normal or variant phenotypes along with their respective mother plants as reference. The numbers of emblings for each variant phenotype are also given.

The somatic embryogenesis process (Figure 1) involved the following stages:

*1) Production of embryogenic callus:* pieces of young leaves were surface-sterilized and used as explants. The explants were cultured for 1 month on a 1/2 strength MS [74] “C” callogenesis medium [75] containing 2.3 µM 2,4-D, 4.9 µM indole-3-butyric acid (IBA) and 9.8 μM iso-pentenyladenine (iP), and then transferred for 6 months to MS/2 “ECP” embryogenic callus production medium [75] containing 4.5 µM 2,4-D and 17.7 µM benzylaminopurine (6-BA). All media were solidified using 2.4 g/l Phytagel (Sigma, Steinheim, Germany). These steps were carried out at 26–27°C in the dark.

*2) 6-month multiplication step and embryo regeneration.* Fully developed somatic embryos were mass regenerated via two distinct multiplication processes, i.e. either secondary (or repetitive) embryogenesis or embryogenic suspensions (Figure 1).

Secondary embryogenesis (SCE). Two hundred mg of embryogenic aggregates were placed in a 1 liter-RITA® temporary immersion bioreactor (CIRAD, Montpellier, France; [76]) along with 200 ml of “R” MS/2 regeneration medium [75] containing 17.76 µM 6-BA, in darkness for 6 weeks. An immersion frequency of 1 min every 12 h was applied. Proliferating embryo masses were then placed in 1/2 strength MS [74] regeneration medium containing 5.6 µM 6-BA and subcultured once every 6 weeks for three proliferation cycles. Secondary embryos were produced with an immersion frequency of 1 min every 12 h and a high culture density (approx. 10 000 embryos). The cultures were kept at 27°C, with a 12 h/12 h photoperiod and 50 µmol m^−2^ s^−1^ photosynthetic photon flux density.

Embryogenic cell suspensions (ESP). Embryogenic calli were transferred to 100 ml Erlenmeyer flasks at a density of 1 g/L in 1/4 MS strength Yasuda liquid proliferation medium [77] with 1.36 µM 2,4-D and 4.4 µM 6-BA. Suspension cultures were maintained by the monthly transfer of 1 g/L of embryogenic aggregates into fresh medium. Six month-old suspensions were used for somatic embryo regeneration. Embryo differentiation was initiated by transferring embryogenic aggregates at a density of 1 g/L in 250 ml Erlenmeyer flasks in a full strength MS medium containing 1.35 µM 6-BA. Fully developed torpedo-shaped embryos were obtained after two 4 week subcultures in such conditions. All suspension cultures were shaken at 110 rpm at 27 °C under 50 µmol m^−2^ s^−1^ photosynthetic photon flux density.

*3) Pre-germination in a bioreactor.* Germination was triggered by applying a low culture density of around 800–900 embryos per 1 l-RITA® bioreactor. An "EG" embryo germination medium [75] containing 1.33 μM BA was used for 2 months and finally, for 2 weeks, the “EG” culture medium was supplemented with 234 mM sucrose. By the end of the in vitro culture stage, each bioreactor contained around 700 pre-germinated cotyledonary somatic embryos with an elongated embryonic axis and a pair of open, chlorophyllous cotyledons. Pre-germination was conducted in the light (12/12 h, 50 µmol m^−2^ s^−1^).

*4) Plantlet conversion* was obtained after direct sowing of mature somatic embryos in the nursery. Mature embryos were sown vertically on top of the substrate (two parts soil, one part sand, one part coffee pulp) sterilized by chemical treatment (Dazomet (DMTT), Union Carbide). The somatic embryo culture density in the plastic boxes (l.w.h  = 30/21/10 cm) was approximately 3600 m^−2^. The cultures were placed under a transparent roof that provided 50% shade, and were watered for 2 min twice daily. Conversion of somatic embryos into plants was generally observed 12 weeks after sowing, and characterized by the emergence of a stem bearing at least two pairs of true leaves.

*5) Growth and hardening in the nursery (21 weeks).* Plantlets grown from somatic embryos were transferred to 0.3 L plugs on a substrate comprising peat-based growing medium (Pro-mix, Premier Tech Ltd, Canada) and coffee pulp (3/1, v/v) under conventional nursery conditions until they reached the required size for planting in the field (approx. 30 cm). During this stage, the shade (50% light interception) and relative humidity (80%) were gradually reduced over 4 weeks to 0% light interception, with natural RH ranging from 65 to 90%.

### Molecular analysis

#### DNA extraction

Young fully expanded leaves were selected on three year-old plants for molecular analysis. Genomic DNA was isolated from 100 mg of lyophilized leaves using Dellaporta buffer [78] containing sodium dodecyl sulfate (SDS) detergent and sodium bisulfite 1% w/v to avoid leaf oxidation. DNA was purified in spin-column plates as described in the DNeasy plant kit protocol from QIAGEN.

#### AFLP markers

AFLP analysis was carried out as described by Vos *et al*. [79], with a total of four primer combinations (Table 2), using 5-FAM or 5-HEX fluorescently labeled *Eco*R1 (+3) and unlabeled *Mse*I (+3) primers. A touchdown PCR program for selective amplification was performed in an Eppendorf thermocycler under the following conditions: 3 min at 94°C, 12 cycles of 45 s at 94°C, 12 cycles of 45 s at 65°C and 1 min at 72°C; the annealing temperature was decreased by 0.7°C per cycle from a starting point of 65°C during this stage, with a final round of 25 cycles of 94°C for 45 s, 56°C for 45 s, 72°C for 1 min and a final elongation step of 72°C for 1 min. The same PCR conditions were found to be appropriate for MSAP in preliminary tests.

#### MSAP markers

MSAP analysis was carried out as described by Reyna-López *et al*. [19] with minor adaptations for capillary electrophoresis. The MSAP protocol is an adaptation of the AFLP method for the evaluation of different states of methylation in the symmetric sequence CCGG. In the MSAP protocol, the frequent cutting endonuclease (*Mse*I) was replaced by the two isoschizomeric restriction enzymes *Hpa*II and *Msp*I with different sensitivity to the methylation state of the symmetric sequence CCGG (Table 4). Specifically, *Hpa*II is able to recognize and cut only when the CCGG sequence is unmethylated or hemi-methylated on the external cytosine. *Msp*I is able to cut when CCGG sequence is unmethylated or if the internal cytosine is fully or hemi-methylated. Both *Hpa*II and *Msp*I are unable to cut if the external cytosine presents full methylation. DNA methylation in plants commonly occurs at cytosine bases in all sequence contexts: the symmetric CG and CHG, in which (H = A, T or C) and the asymmetric CHH contexts [17]. Selective amplification included a total of eight primer combinations per isoschizomer (Table 2). *Hpa*II (+2) primers were fluorescently labeled with 5-FAM or 5-HEX while *Eco*R1 (+3) remained unlabeled. In order to reduce the possibility of technical artifacts, two repetitions using different DNA extractions were performed for each primer combination.

#### Capillary electrophoresis and data analysis

PCR products were separated by capillary electrophoresis with Pop 7^™^ polymer in a 16 capillary 3130 XL Genetic Analyzer from Applied Biosystems using an internally manufactured 524 ROX fluorophore as sizing standard. The fragments used for fingerprinting were visualized as electropherograms in applied Biosystems software GeneMapper® version 3.7. Informative fragments were mostly found in the 100–450 bp range. All amplified fragments were classified based on the primer combination used and their size. The sample fingerprint data was converted to binary code, with “1” denoting the presence of the fragment and “0” the absence. Different binary matrices were constructed for comparative analysis depending on the kind of molecular marker.

As shown in Table 4, the MSAP patterns were classified as follows: Pattern 1 when a comigrating amplified fragment was obtained from the DNA template digested by both restriction enzymes *Hpa*II and *Msp*I; Pattern 2 and 3 when an amplification fragment was obtained only from the DNA template digested by *Hpa*II or *Msp*I, respectively.

Before analysis of the embling versus mother plant population, we successfully verified, on a set of plants from the Caturra variety, that the same MSAP patterns were systematically generated whatever the plant age and the leaf developmental stage (data not shown). Hence, a possible developmental variability in the studied plant material does not seem to introduce any additional source of variation in the methylation state. Nevertheless, in all experiments, only leaves from the same developmental stage were chosen.

#### Slide preparation and karyotyping

Root tips were harvested from individual adult emblings and placed in an aqueous solution of 8-hydroxyquinoline (2.5 mM) used as pre-treatment, for 4 h in darkness (2 h at 4°C plus 2 h at room temperature). A solution of Carnoy (absolute ethanol and glacial acetic acid, 3∶1 v/v) was used to fix the tissues for at least 24 h at −20°C. Fixed material was then stored in 70% ethanol at 4°C until use for slide preparation. The stored root tips were used for slide preparations by employing the technique for cell dissociation of enzymatically macerated roots, as described previously by Herrera *et al.* [80]. Preparations were frozen in liquid nitrogen in order to remove the coverslips, stained with 4',6-diamidino-2-phenylindole, DAPI (1µg/mL), and mounted in Vectashield (Vector Laboratories, Peterborough, UK).

In order to determine the occurrence of chromosome modifications, individual plants of *C. arabica* regenerated by somatic embryogenesis showing a normal (2 plants) or variant (11 plants) phenotype were submitted to karyotype analysis. The Angustifolia, Bullata, Dwarf, Giant and Variegata phenotypic variants were analyzed. During slide examination, mitotic cells at metaphase or prometaphase stages were used for chromosome counting. Between 4 and 8 mitotic cells from each individual were analyzed to determine the chromosome number. The best examples were photomicrographed at metaphase to document the chromosome number and morphology. A Nikon Eclipse 90i epi-fluorescence microscope equipped with a digital, cooled B/W CCD camera (VDS 1300B Vosskühler ®) was used with the appropriate filter (UV-2E/C excitation wavelength 340–380).
